# Intravenous Tobramycin Inhalation for Patients With Advanced Bronchiectasis With Pseudomonas aeruginosa Infection in Home Medical Care: A Report of Two Cases

**DOI:** 10.7759/cureus.62726

**Published:** 2024-06-19

**Authors:** Katsutoshi Ando

**Affiliations:** 1 Respiratory Medicine, Meguro K Home Clinic, Meguro, JPN

**Keywords:** prognosis, tobramycin inhalation, pseudomonas aeruginosa, home medical care, bronchiectasis

## Abstract

Home medical care faces limitations in the number of doctor and nurse visits, availability of medical devices, and economic factors, making daily injections difficult for in-home patients. We describe two cases of advanced bronchiectasis with *Pseudomonas aeruginosa* infection treated with inhaled tobramycin in a home setting, demonstrating clinical effectiveness. Using commercially available empty eye drop containers to prepare an aseptic inhalation solution and nebulizers easily usable at home, our experience suggests that this could be a viable therapeutic alternative in home medical care.

## Introduction

Bronchiectasis is a chronic lung disease characterized by permanent damage to the smaller and medium-sized airways, resulting in a progressive decline in pulmonary function. Approximately 40%-50% of patients experience two or more exacerbations each year, with a 33% hospitalization rate annually [1]. *Pseudomonas aeruginosa* infection is associated with an increased risk of death and exacerbation in patients with bronchiectasis. Anti-pseudomonal antibiotics such as tazobactam/piperacillin, cefepime, and imipenem are commonly used, but most require intravenous administration.

Tobramycin is an aminoglycoside antibiotic with potent antipseudomonal effects. Nebulized tobramycin inhalation solution (TIS; TOBI®, 300 mg twice daily) is indicated for the management of cystic fibrosis with *P. aeruginosa* infection [2]. It provides a high dose of tobramycin to the lungs while maintaining low serum concentrations of the drug, thus reducing the risk of systemic toxicity and contributing to improved lung function and quality of life. Recently, the TORNASOL trial confirmed the efficacy and safety of TIS in patients with noncystic fibrosis bronchiectasis (NCFBE) with *P. aeruginosa* infection [3].

However, in Japan, TIS is not covered by the national insurance system for patients with NCFBE, and it costs approximately 18,000 yen per day at the patient’s own expense (9,045 yen/TOBI® 300 mg); therefore, it is generally not a realistic treatment option. Meanwhile, a few Japanese case reports have indicated the effectiveness of intravenous tobramycin inhalation, which was prepared from intravenous tobramycin products [4-6]. Although using intravenous products of tobramycin addresses the financial burden (586 yen/90 mg), reported cases were experienced only in hospitals, and no reports of its use have been reported in in-home environments. Especially, in home environments, patients often encounter significant challenges when attempting to extract and prepare drug solutions from intravenous ampules independently. Here, we detail two cases with advanced NCFBE and *P. aeruginosa* infections that were successfully stabilized by intravenous tobramycin inhalation in home environments.

## Case presentation

Case 1

**Figure 1:**
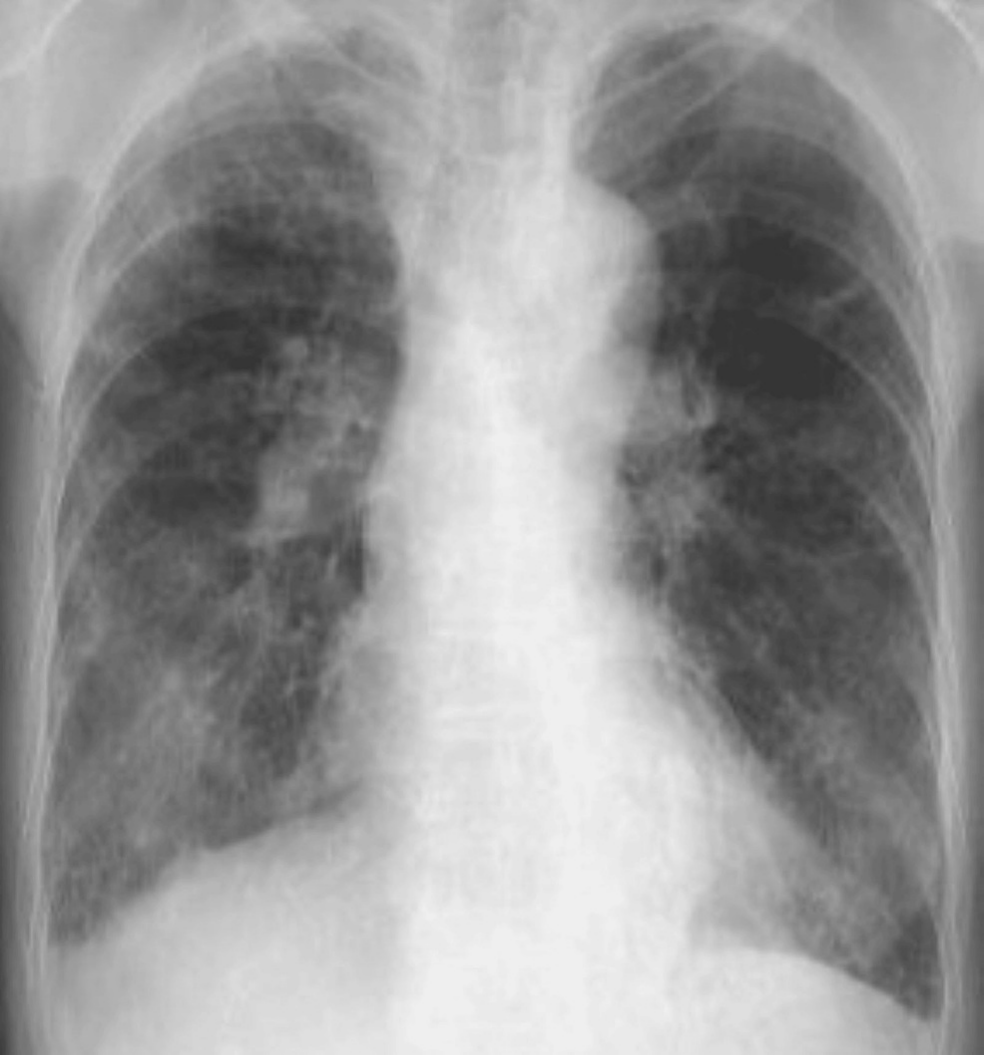
Chest X-ray findings of Case 1. Chest X-ray taken at the hospital showing diffuse bronchiectasis and consolidation in both lung fields.

An 82-year-old female with NCFBE presented with progressive worsening of respiratory symptoms and decreased quality of life with frequent hospitalization. Our home medical care was offered. At 72 years old, she began requiring annual hospitalizations for *P. aeruginosa* pneumonia (Figure 1). Sputum analyses were conducted at each admission, consistently identifying *P. aeruginosa* in culture tests. As she progressed in age and reached 80 years, the frequency of hospitalizations increased by two to three times per year. Within the last two months before the initiation of home medical care, she was hospitalized twice and treated with intravenous cefepime hydrochloride, followed by oral levofloxacin (LVFX). However, her respiratory issues worsened soon after their cessation. This mandates the continuation of oral LVFX for approximately three-fourths of the period after the initiation of home medical care. Therefore, we were concerned about the emergence of LVFX resistance. Owing to the limited availability of oral antibiotics with antipseudomonal effects, we considered treatment with TOBI®, referring to the TORNASOL trial [3], but it was difficult to be given for the insurance noncoverage and the economic issues.

**Figure 2:**
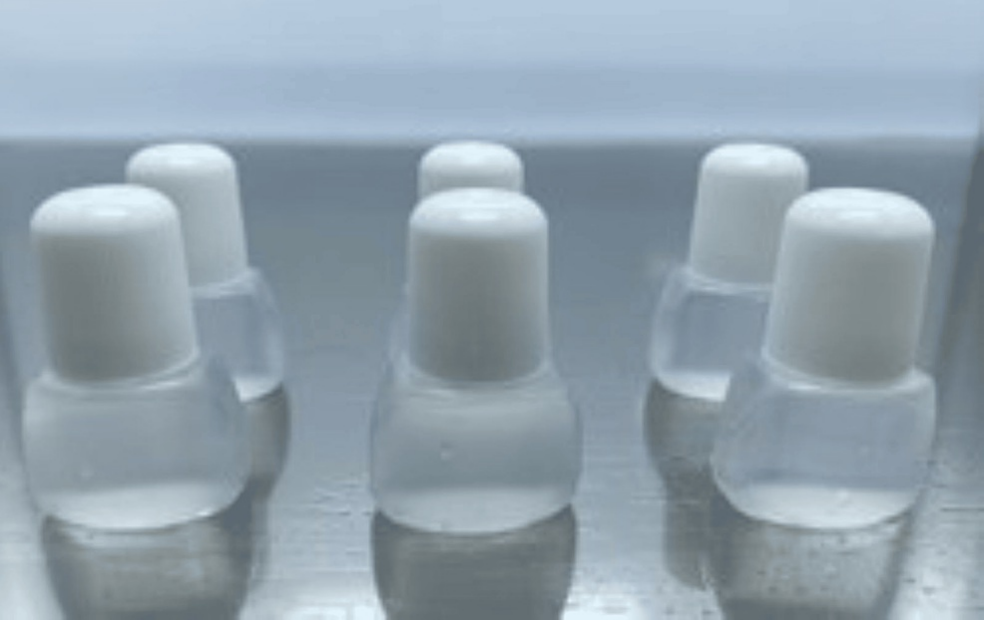
Preparing a nebulized tobramycin inhalation solution. To prepare a nebulized tobramycin inhalation solution in the environment of home medical care, we dispensed two tobramycin ampoules (180 mg) and 1 ml of 0.9% sodium chloride into each commercially available empty eye drop container in a pharmacy sterile cabinet.

We next attempted to use the intravenous tobramycin forms for inhalation, based on the findings of previous studies and case reports [4-6]. However, the method of preparing aseptic inhalation solutions from injection ampoules was an issue in the in-home medical care environment. To address this issue, we utilized commercially available empty eye drop containers (Figure 2) and dispensed two ampoules containing 90 mg of tobramycin (1.5 ml/90 mg) and 1 ml of 0.9% sodium chloride into each container (totaling 4 ml) in a pharmacy sterile cabinet. We prepared seven containers (to cover one week), delivered them to the patient’s house weekly, and stored them in her refrigerator. To assess its efficacy and safety, we used the Chronic Obstructive Pulmonary Disease (COPD) Assessment Test (CAT), which was reported to be a valid, responsive symptom evaluation instrument in bronchiectasis [7], and measured blood tobramycin levels periodically. After agreement with the patient and her family, we initiated intravenous tobramycin inhalation using a commercially available jet nebulizer (Omron NE-C28®, Kyoto, Japan).

**Figure 3:**
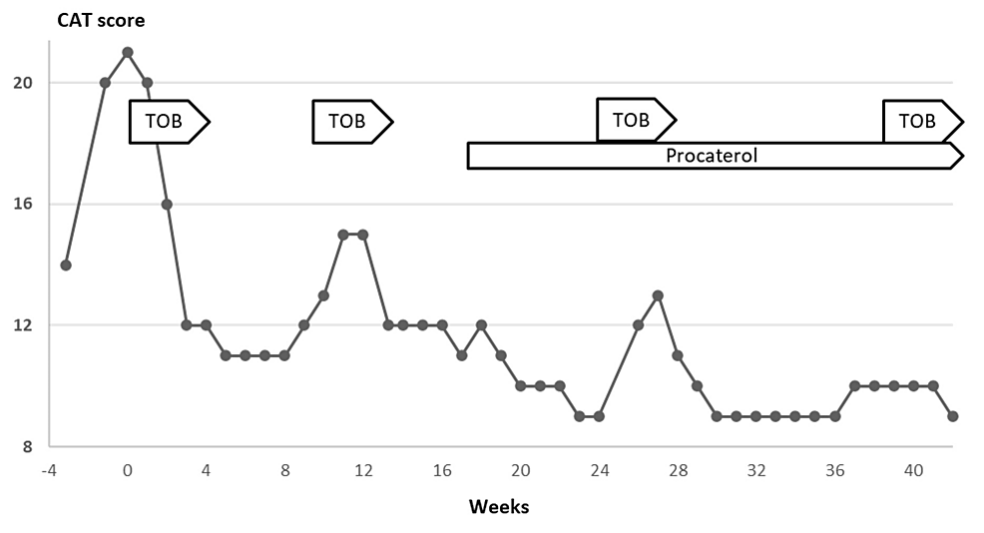
Clinical manifestation of Case 1. Although her CAT score worsened within two weeks after cessation of oral LVFX (from 14 to 21), it rapidly improved after its initiation (from 21 in week 0 to 12 in week three). After the first three weeks of treatment, the patient’s symptoms had been temporarily stabilized, but the CAT score slowly declined from week seven to nine. Then, we additionally used four-week sessions of intravenous tobramycin inhalation (weeks nine to 13) and obtained a good positive impact. From week 17, we started procaterol inhalation with the hope of sputum-clearing and bronchodilatation effects that could extend the period until the next treatment session. After that, she received intravenous tobramycin inhalation in weeks 26 to 30 and weeks 38 to 42, depending on the timing of the worsening of symptoms and CAT score. CAT: Chronic Obstructive Pulmonary Disease Assessment Test; LVFX: levofloxacin; TOB: tobramycin.

As illustrated in Figure 3, her CAT score worsened within two weeks after cessation of oral LVFX but rapidly improved upon starting intravenous tobramycin inhalation. Within three weeks of treatment, her symptoms temporarily stabilized. We additionally used a four-week session of intravenous tobramycin inhalation in weeks nine to 13, weeks 26 to 30, and weeks 38 to 42, contingent upon the onset of symptoms. This regimen significantly improved her respiratory symptoms. Finally, she achieved good general and respiratory conditions with no hospitalization or LVFX use for more than a year, with reported adverse events. No tobramycin was detected in her blood during the period of intravenous tobramycin inhalation.

Case 2

**Figure 4:**
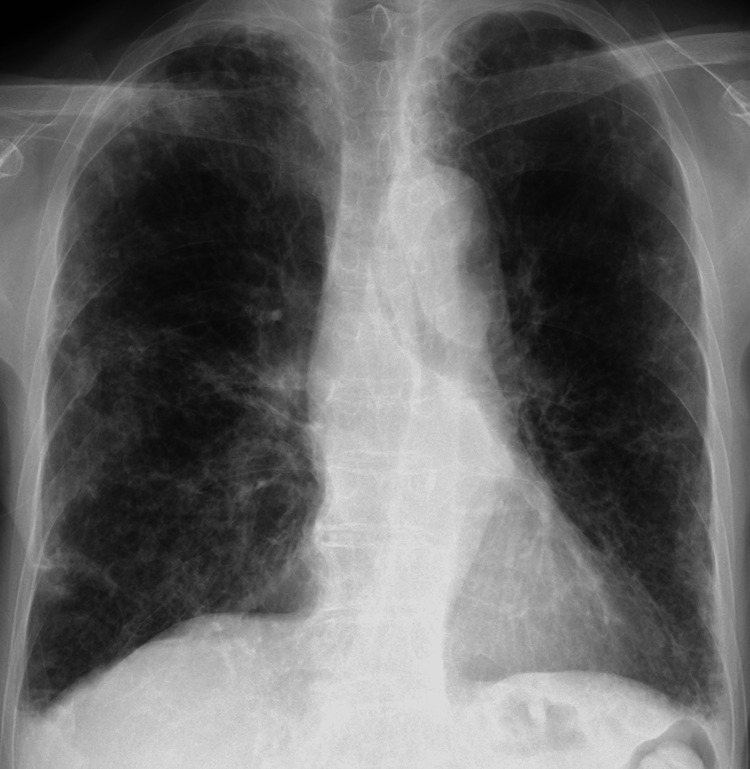
Chest X-ray findings of Case 2. Chest X-ray obtained three months before the initiation of our home medical care showed diffuse bronchiectasis and consolidation.

The second patient with NCFBE was included to exemplify the clinical impact of Case 1. This 85-year-old woman presented with worsening dyspnea and a decline in quality of life due to recurrent *P. aeruginosa* pneumonia. Similar to Case 1, she had received long-term oral LVFX. Once we attempted to cease oral LVFX after the initiation of home medical care, her respiratory issues rapidly worsened (Figure 4). Because she refused hospitalization, daily home visits were required for the intramuscular injection of imipenem (IMP). Her respiratory issues improved but worsened again within two weeks after cessation. Because we had experienced the effectiveness of intravenous tobramycin inhalation in Case 1, we also tried intravenous tobramycin inhalation with the same regimen after her agreement.

**Figure 5:**
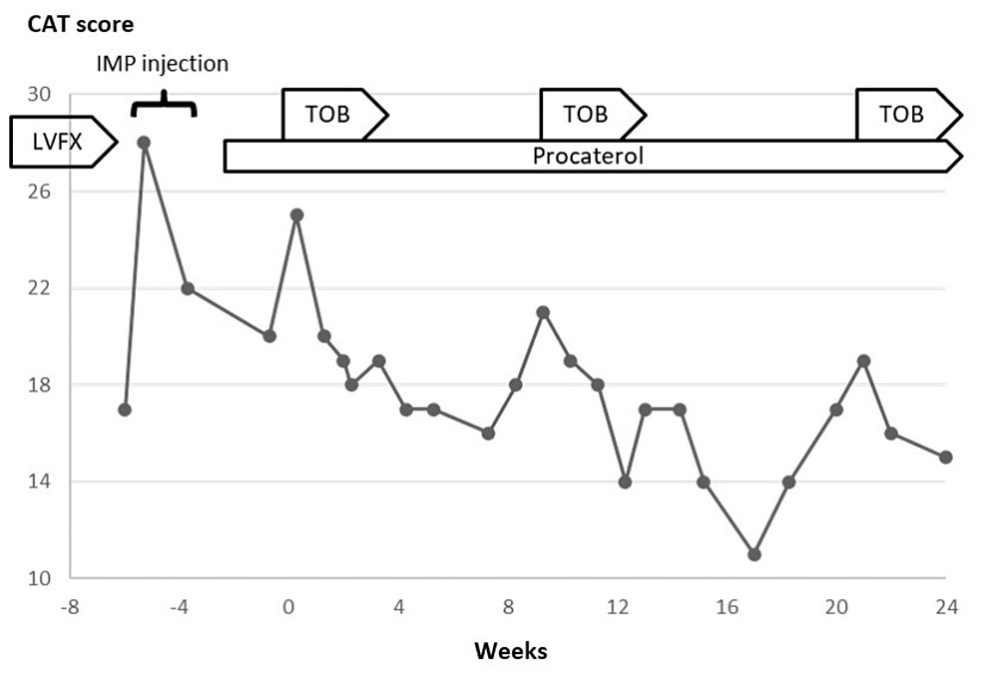
Clinical manifestation of Case 2. After cessation of oral LVFX, her respiratory symptoms rapidly worsened (CAT score increased from 17 to 28), and she needed treatment with imipenem (IMP). After IMP administration, her symptoms lessened with the improvement of her CAT score from 28 to 20, but her respiratory symptoms re-worsened within two weeks after the initiation of IMP treatment (CAT score climbed from 20 to 25). Then, we initiated an additional four weeks of intravenous tobramycin inhalation, and her respiratory symptoms rapidly lessened with the improvement of her CAT score from 25 to 18 (weeks zero to four). We repeated this treatment in weeks nine to 13 and in weeks 20 to 24 depending on the timing of the worsening of symptoms. CAT: Chronic Obstructive Pulmonary Disease Assessment Test; LVFX: levofloxacin; TOB: tobramycin.

After the initiation of intravenous tobramycin inhalation, her respiratory difficulties rapidly subsided, and it was administered for four weeks (weeks zero to four). We used a four-week regimen in weeks nine to 13 and in weeks 20 to 24, depending on the timing of the worsening of symptoms (Figure 5). She significantly improved, with no reported exacerbations, adverse events, or antibiotic use for more than six months. Tobramycin was not detected in her blood during its treatment.

## Discussion

Bronchiectasis is a chronic, progressive lung disease characterized by permanent damage to the smaller and medium-sized airways, resulting in a progressive decline in pulmonary function [1]. With the current progressive increase in the number of patients receiving home medical care in Japan, it is anticipated that the proportion of NCFBE patients receiving home care will increase. However, the need for many doctor and nurse visits, limited availability of medical devices, economic issues, and the preferability and need for specific drug administration methods are challenges for home medical care that must be considered [8]. Thus, the management and prevention of exacerbations are crucial for in-home patients, as are drug administration methods other than injection. The TORNASOL trial suggested that TIS is a promising treatment for NCFBE patients with *P. aeruginosa* infection, but the use of TOBI® poses economic challenges.

This is the first report to evaluate the effectiveness of tobramycin inhalation for in-home medical care of NCFBE patients with *P. aeruginosa* infection. In this environment, the method of preparing an aseptic inhalation solution and inhaling it within a certain time was challenging; however, we overcame these difficulties by using commercially available empty eye drop containers and nebulizers that were easily used at home. Our experience indicates that inhaled tobramycin could be a realistic therapeutic alternative for in-home medical care of advanced bronchiectasis patients with *P. aeruginosa* infection, even at 180 mg of tobramycin doses.

## Conclusions

Using empty eye drop containers to administer inhaled tobramycin effectively managed advanced bronchiectasis in a home care setting, preventing recurrent hospitalizations. This method shows potential as a useful therapeutic alternative for patients with *Pseudomonas aeruginosa* infections. Our cases demonstrated significant improvements in respiratory symptoms without adverse events. Further clinical trials are necessary to confirm the efficacy and safety of this approach.
